# Supplementary material for: How social and economic policies have affected the genome of mezcal agaves: The contrasting stories of Bacanora and Espadín
Source: PLoS One. 2025 Oct 3;20(10):e0324581. doi: 10.1371/journal.pone.0324581 (PMC12494266; doi:10.1371/journal.pone.0324581)
Supplement: S1 Table — (PDF) [file pone.0324581.s001.pdf]

S1 Table. Information for 207 samples of wild and cultivated *Agave angustifolia* from the states of Oaxaca and Sonora.

Management	State	Latitude	Longuitude	Site	N	Data
Cultivated	Oaxaca	16.80641	-96.39257	OaxC_1	3	Klimova et al. 2024 (Unpublished)
Cultivated	Oaxaca	16.809364	-96.309233	OaxC_10	3	Klimova et al. 2023
Cultivated	Oaxaca	16.894628	-96.393919	OaxC_11	3	Klimova et al. 2023
Cultivated	Oaxaca	17.139285	-96.742115	OaxC_12	3	Klimova et al. 2023
Cultivated	Oaxaca	17.219861	-96.801458	OaxC_13	3	Klimova et al. 2023
Cultivated	Oaxaca	17.282948	-96.946815	OaxC_14	4	Klimova et al. 2023
Cultivated	Oaxaca	17.4880291	-97.311609	OaxC_15	3	Klimova et al. 2023
Cultivated	Oaxaca	16.365322	-96.561489	OaxC_2	3	Klimova et al. 2023
Cultivated	Oaxaca	16.40306	-96.950762	OaxC_3	3	Klimova et al. 2023
Cultivated	Oaxaca	16.415992	-96.533222	OaxC_4	4	Klimova et al. 2023
Cultivated	Oaxaca	16.421933	-96.685231	OaxC_5	3	Klimova et al. 2023
Cultivated	Oaxaca	16.45595	-96.952453	OaxC_6	2	Klimova et al. 2023
Cultivated	Oaxaca	16.749128	-96.668237	OaxC_7	3	Klimova et al. 2023
Cultivated	Oaxaca	16.7506175	-96.61702	OaxC_8	3	Klimova et al. 2023
Cultivated	Oaxaca	16.7874891	-96.610789	OaxC_9	4	Klimova et al. 2023
Cultivated	Sonora	28.995333	-109.39328	SonC_1	3	Klimova et al. 2022
Cultivated	Sonora	29.459583	-110.278	SonC_10	3	Klimova et al. 2022
Cultivated	Sonora	27.074972	-109.34483	SonC_11	3	Klimova et al. 2022
Cultivated	Sonora	27.091306	-109.10525	SonC_12	6	Klimova et al. 2022
Cultivated	Sonora	27.042611	-108.98078	SonC_13	3	Klimova et al. 2022
Cultivated	Sonora	28.978472	-109.40067	SonC_2	3	Klimova et al. 2022
Cultivated	Sonora	28.949972	-109.51497	SonC_3	3	Klimova et al. 2022
Cultivated	Sonora	29.210667	-110.12228	SonC_4	3	Klimova et al. 2022
Cultivated	Sonora	29.206333	-110.14303	SonC_5	3	Klimova et al. 2022
Cultivated	Sonora	29.013806	-110.95294	SonC_6	3	Klimova et al. 2022
Cultivated	Sonora	29.022472	-110.95006	SonC_7	3	Klimova et al. 2022
Cultivated	Sonora	29.459583	-110.278	SonC_8	3	Klimova et al. 2022
Cultivated	Sonora	29.685639	-109.63497	SonC_9	3	Klimova et al. 2022
Wild	Oaxaca	16.552574	-95.202035	OaxW_1	3	Klimova et al. 2024 (Unpublished)
Wild	Oaxaca	16.600251	-96.877232	OaxW_10	4	Klimova et al. 2023
Wild	Oaxaca	16.770416	-96.347527	OaxW_11	3	Klimova et al. 2023
Wild	Oaxaca	16.756989	-96.342714	OaxW_12	2	Klimova et al. 2023
Wild	Oaxaca	17.610722	-96.926694	OaxW_13	4	Klimova et al. 2023

Wild	Oaxaca	17.622167	-96.920444	OaxW_14	1	Klimova et al. 2023
Wild	Oaxaca	17.745291	-97.711557	OaxW_15	3	Klimova et al. 2023
Wild	Oaxaca	17.765891	-97.796443	OaxW_16	5	Klimova et al. 2023
Wild	Oaxaca	17.921167	-97.678287	OaxW_17	4	Klimova et al. 2023
Wild	Oaxaca	18.090075	-97.690283	OaxW_18	3	Klimova et al. 2023
Wild	Oaxaca	18.1299331	-97.679669	OaxW_19	4	Klimova et al. 2023
Wild	Oaxaca	16.5263776	-95.189865	OaxW_2	2	Klimova et al. 2024 (Unpublished)
Wild	Oaxaca	16.570895	-94.951545	OaxW_3	4	Klimova et al. 2024 (Unpublished)
Wild	Oaxaca	16.391857	-95.62265	OaxW_4	3	Klimova et al. 2024 (Unpublished)
Wild	Oaxaca	17.2775669	-96.916651	OaxW_5	3	Klimova et al. 2024 (Unpublished)
Wild	Oaxaca	15.931999	-95.769265	OaxW_6	4	Klimova et al. 2024 (Unpublished)
Wild	Oaxaca	16.550173	-95.996978	OaxW_7	4	Klimova et al. 2024 (Unpublished)
Wild	Oaxaca	16.043392	-95.4062	OaxW_8	3	Klimova et al. 2024 (Unpublished)
Wild	Oaxaca	15.721266	-96.474777	OaxW_9	3	Klimova et al. 2024 (Unpublished)
Wild	Sonora	28.960306	-109.59133	SonW_1	3	Klimova et al. 2022
Wild	Sonora	29.770944	-109.72353	SonW_10	3	Klimova et al. 2022
Wild	Sonora	29.803556	-109.63908	SonW_11	3	Klimova et al. 2022
Wild	Sonora	26.736583	-109.56931	SonW_12	3	Klimova et al. 2022
Wild	Sonora	26.768333	-109.47978	SonW_13	3	Klimova et al. 2022
Wild	Sonora	26.81975	-109.28847	SonW_14	4	Klimova et al. 2022
Wild	Sonora	28.477333	-111.04547	SonW_15	3	Klimova et al. 2022
Wild	Sonora	28.820611	-110.55381	SonW_16	2	Klimova et al. 2022
Wild	Sonora	28.567694	-109.69378	SonW_17	3	Klimova et al. 2022
Wild	Sonora	28.488056	-109.36936	SonW_18	2	Klimova et al. 2022
Wild	Sonora	28.432667	-109.24836	SonW_19	2	Klimova et al. 2022
Wild	Sonora	28.947639	-109.50586	SonW_2	3	Klimova et al. 2022
Wild	Sonora	28.94625	-109.66964	SonW_3	3	Klimova et al. 2022
Wild	Sonora	29.184694	-110.15008	SonW_4	3	Klimova et al. 2022
Wild	Sonora	29.167639	-110.15472	SonW_5	4	Klimova et al. 2022
Wild	Sonora	28.303361	-111.40067	SonW_6	3	Klimova et al. 2022
Wild	Sonora	27.977056	-111.13033	SonW_7	3	Klimova et al. 2022
Wild	Sonora	27.940083	-111.09761	SonW_8	3	Klimova et al. 2022
Wild	Sonora	29.492361	-110.19883	SonW_9	3	Klimova et al. 2022
