# Supplementary material for: How social and economic policies have affected the genome of mezcal agaves: The contrasting stories of Bacanora and Espadín
Source: PLoS One. 2025 Oct 3;20(10):e0324581. doi: 10.1371/journal.pone.0324581 (PMC12494266; doi:10.1371/journal.pone.0324581)
Supplement: S2 Table — (PDF) [file pone.0324581.s002.pdf]

S2 Table. Results of BLAST analysis of sequences containing unique alleles for Espadin.

Seq ID	% of identity	Protein Name	Organism
LG01:5028927-5029078	84	Putative polygalacturonase	Ananas comosus (Pineapple) (Ananas ananas)
LG08:45589668-45589819	76	1-phosphatidylinositol 4-kinase (EC 2.7.1.67)	Asparagus officinalis (Garden asparagus)
LG18:62554843-62554994	73.47	1-phosphatidylinositol-3-phosphate 5-kinase (EC 2.7.1.150)	Asparagus officinalis (Garden asparagus)
LG12:22384843-22384994	82	15-cis-phytoene synthase (EC 2.5.1.32)	Asparagus officinalis (Garden asparagus)
LG18:79517793-79517944	79.17	3-oxoacyl-[acyl-carrier-protein] synthase III, chloroplastic	Herrania umbratica
LG03:368925924-368926075	73.58	4-coumarate--CoA ligase (EC 6.2.1.12)	Albuca bracteata (False sea onion) (Ornithogalum longebracteatum)
LG26:69620366-69620517	94	50S ribosomal protein L25-like	Cocos nucifera (Coconut palm)
LG11:1972077-1972228	72	AAA-ATPase At3g50940	Elaeis guineensis var. tenera (Oil palm)
LG03:439768820-439768971	86	ABC transporter C family member 15	Asparagus officinalis (Garden asparagus)
LG25:32661121-32661272	79.59	Adenosine diphosphate glucose pyrophosphorylase small subunit	Agave tequilana (Tequila agave)
LG06:21505318-21505469	69.81	AT-hook motif nuclear-localized protein	Asparagus officinalis (Garden asparagus)
LG15:9378342-9378493	96	BHLH domain-containing protein	Dioscorea zingiberensis
LG22:2620395-2620546	90	C2H2-type domain-containing protein	Asparagus officinalis (Garden asparagus)
LG01:424344716-424344867	72	Cation-transporting P-type ATPase N-terminal domain-containing protein	Davidia involucrata (Dove tree)
LG12:4528115-4528266	90	CCHC-type domain-containing protein	Asparagus officinalis (Garden asparagus)
LG07:98579140-98579291	77.55	Chitin-binding type-1 domain-containing protein	Eucalyptus grandis (Flooded gum)
LG07:98578431-98578582	66.67	chitinase (EC 3.2.1.14)	Protea cynaroides
LG01:203416105-203416256	100	Chlorophyll a-b binding protein, chloroplastic	Asparagus officinalis (Garden asparagus)
LG03:224147954-224148105	89.74	CRAL/TRIO domain	Musa troglodytarum
LG26:2512119-2512270	88	Cytochrome P450 86A1-like	Elaeis guineensis var. tenera (Oil palm)
LG03:169545240-169545391	74.51	Cytochrome P450 CYP81	Narcissus tazetta (Cream narcissus)
LG10:67557331-67557482	68	DDE Tnp4 domain-containing protein	Araucaria cunninghamii (Hoop pine) (Moreton Bay pine)
LG06:28169043-28169194	78.38	Dof-type domain-containing protein	Tripterygium wilfordii (Thunder God vine)
LG01:164295916-164296067	80	DUF641 domain-containing protein	Asparagus officinalis (Garden asparagus)
LG02:348994429-348994580	75	DYW domain-containing protein	Tetracentron sinense (Spur-leaf)

LG19:21152185-21152336	100	Exostosin GT47 domain-containing protein	Ensete ventricosum (Abyssinian banana) (Musa ensete)
LG17:1349962-1350113	88.24	F-box domain-containing protein	Ananas comosus var. bracteatus
LG15:91613035-91613186	95.92	FACT complex subunit	Asparagus officinalis (Garden asparagus)
LG03:463523399-463523550	56.6	Fe2OG dioxygenase domain-containing protein	Aegilops tauschii subsp. strangulata (Goatgrass)
LG20:67307997-67308148	84	Fibronectin type III-like domain-containing protein	Rhynchospora breviuscula
LG02:360749716-360749867	73.47	Flavonoid 3',5'-hydroxylase (EC 1.14.13.21)	Apostasia shenzhenica
LG16:94907033-94907184	86	FLZ-type domain-containing protein	Asparagus officinalis (Garden asparagus)
LG16:58899584-58899735	69.39	Galactose oxidase-like Early set domain-containing protein	Asparagus officinalis (Garden asparagus)
LG14:92307251-92307402	86	General transcription factor 3C polypeptide 3	Asparagus officinalis (Garden asparagus)
LG02:375528889-375529040	72.92	Glucose-methanol-choline oxidoreductase N-terminal domain-containing protein	Asparagus officinalis (Garden asparagus)
LG01:193252288-193252439	68.63	Hexosyltransferase (EC 2.4.1.-)	Musa acuminata subsp. malaccensis (Wild banana) (Musa malaccensis)
LG05:348836397-348836548	89.58	Importin N-terminal domain-containing protein	Asparagus officinalis (Garden asparagus)
LG15:84692642-84692793	69.39	LOW QUALITY PROTEIN: putative E3 ubiquitin-protein ligase RF298	Nicotiana sylvestris (Wood tobacco) (South American tobacco)
LG18:16555810-16555961	64.15	LysM domain-containing protein	Oryza nivara (Indian wild rice) (Oryza sativa f. spontanea)
LG06:42994529-42994680	82.22	Methionine synthase	Trifolium medium
LG25:67613451-67613602	88	Methyltransferase (EC 2.1.1.-)	Asparagus officinalis (Garden asparagus)
LG17:3006998-3007149	82	Mitochondrial carnitine/acylcarnitine carrier-like protein	Gossypium davidsonii (Davidson's cotton) (Gossypium klotzschianum subsp. davidsonii)
LG08:124130041-124130192	91.49	Mitochondrial carrier protein CoAc2 isoform X4	Phoenix dactylifera (Date palm)
LG07:5815119-5815270	72.73	Morc S5 domain-containing protein	Microthlaspi erraticum
LG07:168367262-168367413	75.51	non-specific serine/threonine protein kinase (EC 2.7.11.1)	Cuscuta europaea (European dodder)
LG03:365626565-365626716	98	noroxomaritidine synthase (EC 1.14.19.50)	Asparagus officinalis (Garden asparagus)
LG21:64204196-64204347	92	Oxysterol-binding protein	Arundo donax (Giant reed) (Donax arundinaceus)
LG05:7611576-7611727	93.88	Pectinesterase (EC 3.1.1.11)	Musa troglodytarum
LG24:58145598-58145749	80	Pentatricopeptide repeat-containing protein (EC 3.6.1.-)	Apostasia shenzhenica
LG08:7155423-7155574	67.35	Pentatricopeptide repeat-containing protein At4g21065	Elaeis guineensis var. tenera (Oil palm)
LG10:57945877-57946028	66	Pentatricopeptide repeat-containing protein DOT4, chloroplastic	Cocos nucifera (Coconut palm)

LG06:128793640-128793791	86	Peptidase S8/S53 domain-containing protein	Ensete ventricosum (Abyssinian banana) (Musa ensete)
LG25:67770624-67770775	92	Phosphoglycerate kinase (EC 2.7.2.3)	Asparagus officinalis (Garden asparagus)
LG01:447248743-447248894	98	phosphoribosylformylglycinamidine synthase (EC 6.3.5.3) (Formylglycinamide ribonucleotide amidotransferase) (Formylglycinamide ribotide amidotransferase)	Cocos nucifera (Coconut palm)
LG20:2151319-2151470	72	Plasmodesmata-located protein 8	Phoenix dactylifera (Date palm)
LG05:236039461-236039612	76.09	Probable indole-3-acetic acid-amido synthetase GH3.8	Elaeis guineensis var. tenera (Oil palm)
LG26:12640101-12640252	67.35	procollagen-proline 4-dioxygenase (EC 1.14.11.2)	Musa troglodytarum
LG03:320530733-320530884	82.61	Prolamin-like domain-containing protein	Asparagus officinalis (Garden asparagus)
LG19:68006496-68006647	86	Protein CANDIDATE G-PROTEIN COUPLED RECEPTOR 7-like	Phoenix dactylifera (Date palm)
LG05:355072404-355072555	68.75	Protein DELAY OF GERMINATION 1-like	Punica granatum (Pomegranate)
LG10:11055786-11055937	85.11	Protein DETOXIFICATION	Ensete ventricosum (Abyssinian banana) (Musa ensete)
LG15:11237346-11237497	94	Protein kinase domain-containing protein	Dioscorea zingiberensis
LG02:299930174-299930325	70.21	Protein TIC 22-like, chloroplastic	Asparagus officinalis (Garden asparagus)
LG02:182091071-182091222	94	Protein transport protein SEC24	Cocos nucifera (Coconut palm)
LG06:166335687-166335838	85.11	protein-serine/threonine phosphatase (EC 3.1.3.16)	Kalanchoe fedtschenkoi (Lavender scallops) (South American air plant)
LG06:185997048-185997199	66.67	PUM-HD domain-containing protein	Asparagus officinalis (Garden asparagus)
LG02:458288057-458288208	76	Putative Cytochrome P450 84A1	Cocos nucifera (Coconut palm)
LG16:87417422-87417573	83.87	Putative NO-associated protein 1, chloroplastic/mitochondrial	Cocos nucifera (Coconut palm)
LG08:64652044-64652195	75.68	Putative tocopherol cyclase, chloroplastic	Anthurium amnicola
LG09:39955441-39955592	96	Quinonprotein alcohol dehydrogenase-like	Medicago truncatula (Barrel medic) (Medicago tribuloides)
LG21:36667128-36667279	85.71	RING-type domain-containing protein	Cocos nucifera (Coconut palm)
LG06:63644762-63644913	76	RING-type E3 ubiquitin transferase (EC 2.3.2.27)	Asparagus officinalis (Garden asparagus)
LG23:5701615-5701766	86.11	RNA helicase (EC 3.6.4.13)	Hevea brasiliensis (Para rubber tree) (Siphonia brasiliensis)
LG06:19869556-19869707	72.88	Selenoprotein O	Asparagus officinalis (Garden asparagus)
LG10:57585248-57585399	62.5	Shikimate O-hydroxycinnamoyltransferase	Opuntia streptacantha (Prickly pear cactus) (Opuntia cardona)
LG25:79890595-79890746	71.43	Transcription factor MYB93	Elaeis guineensis var. tenera (Oil palm)
LG09:92132947-92133098	60	Transcriptional regulator of RNA polII, SAGA, subunit	Dendrobium nobile (Orchid)

LG01:209420291-209420442	96.88	Transducin family protein / WD-40 repeat family protein	Zea mays (Maize)
LG15:88501297-88501448	96	type I protein arginine methyltransferase (EC 2.1.1.319)	Phoenix dactylifera (Date palm)
LG07:153277405-153277556	76	U-box domain-containing protein (EC 2.3.2.27) (RING-type E3 ubiquitin transferase PUB)	Anthurium amnicola
LG03:419384969-419385120	77.55	UBX domain-containing protein	Dendrobium nobile (Orchid)
LG10:57585690-57585841	61.54	Uncharacterized acetyltransferase At3g50280	Elaeis guineensis var. tenera (Oil palm)
LG21:13461968-13462119	88.89	WAT1-related protein	Asparagus officinalis (Garden asparagus)
LG09:73410047-73410198	72	WRKY domain-containing protein	Asparagus officinalis (Garden asparagus)
LG03:364304732-364304883	72.92	xyloglucan:xyloglucosyl transferase (EC 2.4.1.207)	Ensete ventricosum (Abyssinian banana) (Musa ensete)
