# Supplementary material for: How social and economic policies have affected the genome of mezcal agaves: The contrasting stories of Bacanora and Espadín
Source: PLoS One. 2025 Oct 3;20(10):e0324581. doi: 10.1371/journal.pone.0324581 (PMC12494266; doi:10.1371/journal.pone.0324581)
Supplement: S3 Table — (PDF) [file pone.0324581.s003.pdf]

**S3 Table.** Results of BLAST analysis of sequences containing unique alleles for cultivated Sonoran samples.

| Seq ID | % of identity | Protein Name | Organism |
|---|---|---|---|
| LG12:22384843-22384994 | 82 | 15-cis-phytoene synthase (EC 2.5.1.32) | Asparagus officinalis (Garden asparagus) |
| LG03:318311075-318311226 | 100 | 26S proteasome non-atpase regulatory subunit | Musa troglodytarum |
| LG18:79517793-79517944 | 79.17 | 3-oxoacyl-[acyl-carrier-protein] synthase III, chloroplastic | Herrania umbratica |
| LG26:69620366-69620517 | 94 | 50S ribosomal protein L25-like | Cocos nucifera (Coconut palm) |
| LG06:49350627-49350778 | 100 | AAA+ ATPase domain-containing protein | Cocos nucifera (Coconut palm) |
| LG17:83594756-83594907 | 88.24 | ABC transporter B family member 1 | Elaeis guineensis var. tenera (Oil palm) |
| LG16:6974204-6974355 | 90 | ABC transporter domain-containing protein | Asparagus officinalis (Garden asparagus) |
| LG10:69892604-69892755 | 64 | Acetyltransferase | Asparagus officinalis (Garden asparagus) |
| LG14:93841709-93841860 | 83.78 | Alpha/beta hydrolase fold-3 domain-containing protein | Musa balbisiana (Banana) |
| LG12:88532897-88533048 | 74.36 | Aluminum-activated malate transporter 10 | Artemisia annua (Sweet wormwood) |
| LG04:299109553-299109704 | 100 | Amino acid permease 3 | Cinnamomum micranthum f. kanehirae |
| LG19:57209081-57209232 | 86.84 | AP2/ERF domain-containing protein | Asparagus officinalis (Garden asparagus) |
| LG28:36577429-36577580 | 82.35 | Armadillo repeat-containing domain-containing protein | Asparagus officinalis (Garden asparagus) |
| LG26:19169049-19169200 | 93.75 | Arogenate dehydratase (EC 4.2.1.91) | Asparagus officinalis (Garden asparagus) |
| LG05:4077133-4077284 | 98 | Autophagy-related protein 18a | Musa balbisiana (Banana) |
| LG02:7379986-7380137 | 97.96 | Auxin response factor | Asparagus officinalis (Garden asparagus) |
| LG10:70260951-70261102 | 68.33 | Basic leucine zipper 19-like | Ananas comosus (Pineapple) (Ananas ananas) |
| LG17:33464803-33464954 | 94 | Beta-catenin-like protein 1 N-terminal domain-containing protein | Vanilla planifolia (Vanilla) |
| LG15:9378342-9378493 | 96 | BHLH domain-containing protein | Dioscorea zingiberensis |
| LG19:61595550-61595701 | 92 | Bromo domain-containing protein | Asparagus officinalis (Garden asparagus) |
| LG14:67901652-67901803 | 97.06 | BTB/POZ domain-containing protein POB1-like | Phoenix dactylifera (Date palm) |
| LG19:68407917-68408068 | 70.83 | C2H2-type domain-containing protein | Zizania palustris (Northern wild rice) |
| LG25:63052655-63052806 | 76.19 | C3H1-type domain-containing protein | Asparagus officinalis (Garden asparagus) |

| | | | |
|---|---|---|---|
| LG26:44255730-44255881 | 81.82 | CASP-like protein | Asparagus officinalis (Garden asparagus) |
| LG02:362567112-362567263 | 100 | catalase (EC 1.11.1.6) | Hemerocallis sp. (Daylily) |
| LG03:300307012-300307163 | 100 | Cellulose synthase A catalytic subunit 4 | Trifolium pratense (Red clover) |
| LG06:69440537-69440688 | 72 | Centromere-associated protein E-like isoform X2 | Phoenix dactylifera (Date palm) |
| LG21:60382666-60382817 | 83.67 | Chalcone-flavonone isomerase family protein | Narcissus tazetta subsp. chinensis |
| LG21:47638586-47638737 | 92 | Cinnamate-4-hydroxylase | Actinidia deliciosa (Kiwi) |
| LG11:44079613-44079764 | 90.7 | CNH domain-containing protein | Asparagus officinalis (Garden asparagus) |
| LG03:224147954-224148105 | 89.74 | CRAL/TRIO domain | Musa troglodytarum |
| LG23:55460728-55460879 | 78.38 | Cyclin C-terminal domain-containing protein | Ensete ventricosum (Abyssinian banana) (Musa ensete) |
| LG09:18283086-18283237 | 72.34 | Cyclin-like domain-containing protein | Colocasia esculenta (Wild taro) (Arum esculentum) |
| LG03:169545351-169545502 | 81.63 | Cytochrome P450 CYP81 | Narcissus tazetta (Cream narcissus) |
| LG12:98819013-98819164 | 88 | Cytosine-specific methyltransferase (EC 2.1.1.37) | Dioscorea zingiberensis |
| LG10:67557331-67557482 | 68 | DDE Tnp4 domain-containing protein | Araucaria cunninghamii (Hoop pine) (Moreton Bay pine) |
| LG01:76391726-76391877 | 78.57 | DnaJ subfamily b member 13-like protein | Trifolium pratense (Red clover) |
| LG19:67184801-67184952 | 83.78 | DUF4005 domain-containing protein | Asparagus officinalis (Garden asparagus) |
| LG01:277899644-277899795 | 70 | DUF538 family protein | Papaver nudicaule (Iceland poppy) |
| LG04:7990063-7990214 | 78 | DUF641 domain-containing protein | Asparagus officinalis (Garden asparagus) |
| LG24:56357033-56357184 | 78 | ENTH domain-containing protein | Asparagus officinalis (Garden asparagus) |
| LG16:90391449-90391600 | 67.35 | Extra-large guanine nucleotide-binding protein 3 | Dioscorea zingiberensis |
| LG17:1349962-1350113 | 88.24 | F-box domain-containing protein | Ananas comosus var. bracteatus |
| LG15:92629633-92629784 | 92 | FACT complex subunit | Asparagus officinalis (Garden asparagus) |
| LG03:463523399-463523550 | 56.6 | Fe2OG dioxygenase domain-containing protein | Aegilops tauschii subsp. strangulata (Goatgrass) |
| LG03:311933416-311933567 | 84 | Flavin-containing monooxygenase (EC 1.-.-.-) | Asparagus officinalis (Garden asparagus) |
| LG17:6794741-6794892 | 81.63 | Fungal lipase-type domain-containing protein | Dioscorea zingiberensis |
| LG16:58899584-58899735 | 69.39 | Galactose oxidase-like Early set domain-containing protein | Asparagus officinalis (Garden asparagus) |
| LG23:1957334-1957485 | 79.59 | GCF C-terminal domain-containing protein | Vanilla planifolia (Vanilla) |
| LG06:55800407-55800558 | 78 | GDSL esterase/lipase 7 | Dioscorea zingiberensis |

| | | | |
|---|---|---|---|
| LG01:421727593-421727744 | 92 | Gem-associated protein 2 | Asparagus officinalis (Garden asparagus) |
| LG29:30404636-30404787 | 75 | H(+)-exporting diphosphatase (EC 7.1.3.1) | Rhamnella rubrinervis |
| LG12:24530962-24531113 | 80.85 | Hexosyltransferase (EC 2.4.1.-) | Camellia sinensis var. sinensis |
| LG12:84664327-84664478 | 93.88 | Homeobox domain-containing protein | Dioscorea zingiberensis |
| LG16:84726936-84727087 | 78 | Homeobox-leucine zipper ANTHOCYANINLESS 2 | Olea europaea subsp. europaea |
| LG05:348836397-348836548 | 89.58 | Importin N-terminal domain-containing protein | Asparagus officinalis (Garden asparagus) |
| LG02:140864441-140864592 | 73.91 | Integrase catalytic domain-containing protein | Hibiscus trionum (Flower of an hour) |
| LG25:69297615-69297766 | 80 | Integrator complex subunit 3 | Phoenix dactylifera (Date palm) |
| LG17:18639229-18639380 | 95.24 | LisH domain-containing protein | Asparagus officinalis (Garden asparagus) |
| LG06:108456416-108456567 | 96.97 | LOB domain-containing protein | Rhododendron williamsianum |
| LG03:94470710-94470861 | 82 | Major facilitator superfamily (MFS) profile domain-containing protein | Ensete ventricosum (Abyssinian banana) (Musa ensete) |
| LG03:373534160-373534311 | 96 | Major facilitator superfamily (MFS) profile domain-containing protein | Punica granatum (Pomegranate) |
| LG25:67613451-67613602 | 88 | Methyltransferase (EC 2.1.1.-) | Asparagus officinalis (Garden asparagus) |
| LG26:74839213-74839364 | 92 | Mon2/Sec7/BIG1-like HUS domain-containing protein | Kingdonia uniflora |
| LG03:281056181-281056332 | 82 | Myosin motor domain-containing protein | Asparagus officinalis (Garden asparagus) |
| LG18:14720680-14720831 | 93.88 | NAC domain-containing protein 21/22 | Elaeis guineensis var. tenera (Oil palm) |
| LG25:19964303-19964454 | 82 | NADPH-dependent aldehyde reductase-like protein, chloroplastic | Asparagus officinalis (Garden asparagus) |
| LG22:52695174-52695325 | 75 | NEDD8 ultimate buster 1 | Anisodus acutangulus |
| LG11:1807790-1807941 | 95.35 | non-specific serine/threonine protein kinase (EC 2.7.11.1) | Asparagus officinalis (Garden asparagus) |
| LG03:408760898-408761049 | 96 | Os03g0245700 protein | Oryza sativa subsp. japonica (Rice) |
| LG02:413616970-413617121 | 66 | Pectinesterase (EC 3.1.1.11) | Papaver nudicaule (Iceland poppy) |
| LG02:425795501-425795652 | 84.78 | Pentacotripeptide-repeat region of PRORP domain-containing protein | Asparagus officinalis (Garden asparagus) |
| LG04:223513561-223513712 | 73.47 | Pentatricopeptide repeat-containing protein | Musa balbisiana (Banana) |
| LG01:121819847-121819998 | 72 | Pentatricopeptide repeat-containing protein At3g61360 | Elaeis guineensis var. tenera (Oil palm) |
| LG18:66361694-66361845 | 73.47 | Pentatricopeptide repeat-containing protein, chloroplastic | Ananas comosus (Pineapple) (Ananas ananas) |
| LG01:67158723-67158874 | 83.67 | Peptidase C1A papain C-terminal domain-containing protein | Asparagus officinalis (Garden asparagus) |
| LG06:128793640-128793791 | 86 | Peptidase S8/S53 domain-containing protein | Ensete ventricosum (Abyssinian banana) (Musa ensete) |

| | | | |
|---|---|---|---|
| LG05:429603378-429603529 | 76.19 | Phospholipid-transporting ATPase (EC 7.6.2.1) | Ananas comosus (Pineapple) (Ananas ananas) |
| LG01:447248743-447248894 | 98 | phosphoribosylformylglycinamidine synthase (EC 6.3.5.3) (Formylglycinamide ribonucleotide amidotransferase) (Formylglycinamide ribotide amidotransferase) | Cocos nucifera (Coconut palm) |
| LG09:69050450-69050601 | 75.51 | Piriformospora indica-insensitive protein 2 | Ananas comosus (Pineapple) (Ananas ananas) |
| LG01:61759602-61759753 | 65.31 | Plasma membrane ATPase (EC 7.1.2.1) | Daucus carota subsp. sativus (Carrot) |
| LG20:2151319-2151470 | 72 | Plasmodesmata-located protein 8 | Phoenix dactylifera (Date palm) |
| LG30:36123974-36124125 | 76 | Potassium transporter | Carex littledalei |
| LG07:148450652-148450803 | 82.61 | Probable ubiquitin carboxyl-terminal hydrolase MINDY-4 (EC 3.4.19.12) (Deubiquitinating enzyme MINDY-3) (Probable deubiquitinating enzyme MINDY-4) | Vanilla planifolia (Vanilla) |
| LG01:127438686-127438837 | 90 | Probable xyloglucan glycosyltransferase 5 | Elaeis guineensis var. tenera (Oil palm) |
| LG11:5581161-5581312 | 89.47 | Proteasome subunit alpha type-4-A | Striga hermonthica (Purple witchweed) (Buchnera hermonthica) |
| LG15:11237060-11237211 | 89.47 | Protein kinase domain-containing protein | Arachis hypogaea (Peanut) |
| LG02:182091071-182091222 | 94 | Protein transport protein SEC24 | Cocos nucifera (Coconut palm) |
| LG26:79448814-79448965 | 93.88 | Pterin-binding domain-containing protein | Leersia perrieri |
| LG03:358252824-358252975 | 92 | Putative dead box ATP-dependent RNA helicase | Hibiscus syriacus (Rose of Sharon) |
| LG01:5028927-5029078 | 84 | Putative polygalacturonase | Ananas comosus (Pineapple) (Ananas ananas) |
| LG08:64652044-64652195 | 75.68 | Putative tocopherol cyclase, chloroplastic | Anthurium amnicola |
| LG03:74638595-74638746 | 81.08 | Reticulon-like protein | Colocasia esculenta (Wild taro) (Arum esculentum) |
| LG26:38394025-38394176 | 90 | RING-type domain-containing protein | Asparagus officinalis (Garden asparagus) |
| LG15:16634587-16634738 | 81.25 | RING-type E3 ubiquitin transferase (EC 2.3.2.27) | Dioscorea zingiberensis |
| LG06:15097513-15097664 | 78.72 | RRM domain-containing protein | Asparagus officinalis (Garden asparagus) |
| LG12:13572823-13572974 | 80.95 | S1 motif domain-containing protein | Asparagus officinalis (Garden asparagus) |
| LG05:355287738-355287889 | 79.59 | SANT domain-containing protein | Asparagus officinalis (Garden asparagus) |
| LG24:57584309-57584460 | 91.67 | Serine/threonine protein phosphatase 2A regulatory subunit | Cinnamomum micranthum f. kanehirae |
| LG01:368594741-368594892 | 88.46 | SHSP domain-containing protein | Asparagus officinalis (Garden asparagus) |
| LG26:92893242-92893393 | 96 | Signal responsive 1 | Actinidia rufa |
| LG15:88501297-88501448 | 96 | type I protein arginine methyltransferase (EC 2.1.1.319) | Phoenix dactylifera (Date palm) |

| | | | |
|---|---|---|---|
| LG01:159124530-159124681 | 86.49 | Ubiquitin-like modifier-activating enzyme ATG7 (Autophagy-related protein 7) | Elaeis guineensis var. tenera (Oil palm) |
| LG30:11362748-11362899 | 98 | UBR-type domain-containing protein | Asparagus officinalis (Garden asparagus) |
| LG25:42415863-42416014 | 70 | UDP-glycosyltransferases domain-containing protein | Asparagus officinalis (Garden asparagus) |
