# Supplementary material for: How social and economic policies have affected the genome of mezcal agaves: The contrasting stories of Bacanora and Espadín
Source: PLoS One. 2025 Oct 3;20(10):e0324581. doi: 10.1371/journal.pone.0324581 (PMC12494266; doi:10.1371/journal.pone.0324581)
Supplement: S1 Fig — Bar plots of the individual assignment probabilities (vertical axis) for the best number of genetic clusters from K = 3 inferred using FastStructure. (PDF) [file pone.0324581.s004.pdf]

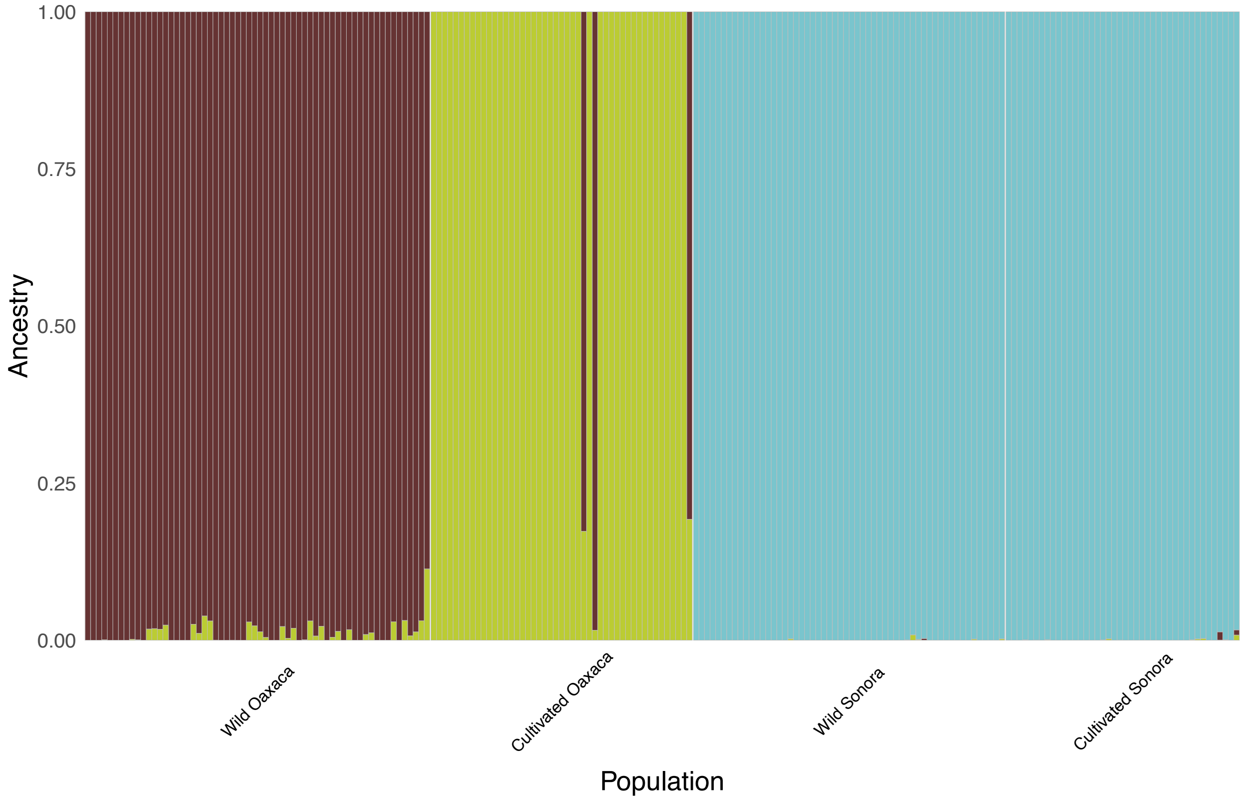

S1 Figure. Population genetic structure of the 207 wild and cultivated *A. angustifolia* individuals. Bar plots of the individual assignment probabilities (vertical axis) for the best number of genetic clusters from $K = 3$ inferred using FastStructure.
